# The prediction of survival in Gastric Cancer based on a Robust 13-Gene Signature

**DOI:** 10.7150/jca.49658

**Published:** 2021-04-12

**Authors:** Guoguang Wang, Tian Zhan, Fan Li, Jian Shen, Xiang Gao, Lei Xu, Yuan Li, Jianping Zhang

**Affiliations:** 1 Department of General Surgery, The Second Affiliated Hospital of Nanjing Medical University, Nanjing, China.; 2 Jiangsu Key Lab of Cancer Biomarkers, Prevention and Treatment, Jiangsu Collaborative Innovation Center For Cancer Personalized Medicine, School of Public Health, Nanjing Medical University, Nanjing, China.

**Keywords:** drug resistance, gastric cancer, Kyoto Encyclopedia of Genes and Genomes pathway, recurrence, prognostic signature

## Abstract

Gastric cancer represents a major public health problem. Owing to the great heterogeneity of GC, conventional clinical characteristics are limited in the accurate prediction of individual outcomes and survival. This study aimed to establish a robust gene signature to predict the prognosis of GC based on multiple datasets. Initially, we downloaded raw data from four independent datasets of The Cancer Genome Atlas (TCGA) and Gene Expression Omnibus (GEO), and performed univariate Cox proportional hazards regression analysis to identify prognostic genes associated with overall survival (OS) from each dataset. Thirteen common genes from four datasets were screened as candidate prognostic signatures. Then, a risk score model was developed based on this 13‑gene signature and validated by four independent datasets and the entire cohort. Patients with a high-risk score had poorer OS and recurrence-free survival (RFS). Multivariate regression and stratified analysis revealed that the 13-gene signature was not only an independent predictive factor but also associated with recurrence when adjusting for other clinical factors. Furthermore, in the high-risk group, gene set enrichment analysis (GSEA) showed that the mTOR signaling pathway and MAPK signaling pathway were significantly enriched. The present study provided a robust and reliable gene signature for prognostic prediction of both OS and RFS of patients with GC, which may be useful for delivering individualized management of patients.

## Introduction

GC remains a major challenge for public health worldwide 1. Accurate prediction of prognosis can confirm patients who would benefit from more radical treatment, such as chemotherapy, neoadjuvant therapy and targeted molecular therapy. Currently, the tumor-node-metastasis (TNM) classification system is still the most common method to select therapeutic strategies and evaluate the prognosis of patients with GC 2. Nevertheless, various outcomes have been identified in patients with GC with similar clinical factors, which suggests that the predictive efficacy of conventional models may be insufficient 3-5. Hence, it is crucial to develop robust and reliable prognostic signatures to improve individualized survival predictions in GC.

Modern biomedical research, such as microarray and next-generation sequencing analyses, has explored many GC prognostic genes that are crucial for risk stratification and personalized treatment decisions 6, 7. However, the vast majority of studies have concentrated mainly on a single gene, and its predictive ability is insufficient compared with multiple biomarker-based models 8, 9. In clinical practice, accurately predicting OS for patients with GC may facilitate individual clinical decision-making. In this study, we established a robust 13-gene prognostic signature for gastric cancer by integrating multiple datasets, which might complement classical clinical prognostic characteristics, and further aid clinicians in personalized treatment planning.

## Materials and methods

### Acquisition of gene expression clinical data

Three independent datasets and corresponding clinical information were downloaded from GEO (GEO; https://www.ncbi.nlm.nih.gov/geo/), under the accession numbers GSE15459, GSE62254 and GSE57303, and one dataset in TCGA was employed from University of California Santa Cruz Xenabrowser (UCSC Xena) (http://genome.ucsc.edu/). The mRNA expression profiles of the GSE15459, GSE62254 and GSE57303 datasets were all detected by using the GPL570 platform (Affymetrix Human Genome U133 Plus 2.0 Array). After the samples with overall survival (OS) ≤ 30 days were excluded, 884 patients were enrolled, including 182 samples from GSE15459, 299 from GSE62254, 70 from GSE57303, and 333 from TCGA (Table S1). In terms of cancer recurrence, patients from TCGA were excluded if (i) the records of primary therapy outcome and radiation therapy were not clearly presented (n = 26); (ii) patients underwent radiation therapy (n = 45); or (iii) patients with stage I gastric cancer who recurred within one year did not achieve complete resection after primary therapy (n = 2), resulting in the enrolling of a total of 191 patients (Table S2). Raw datasets from the GEO database were normalized respectively via Robust Multichip Average (RMA) in the affy package of R, while the TCGA data were normalized as transcripts per million (TPM).

### Development and validation of the gene signature

**Figure 1:**
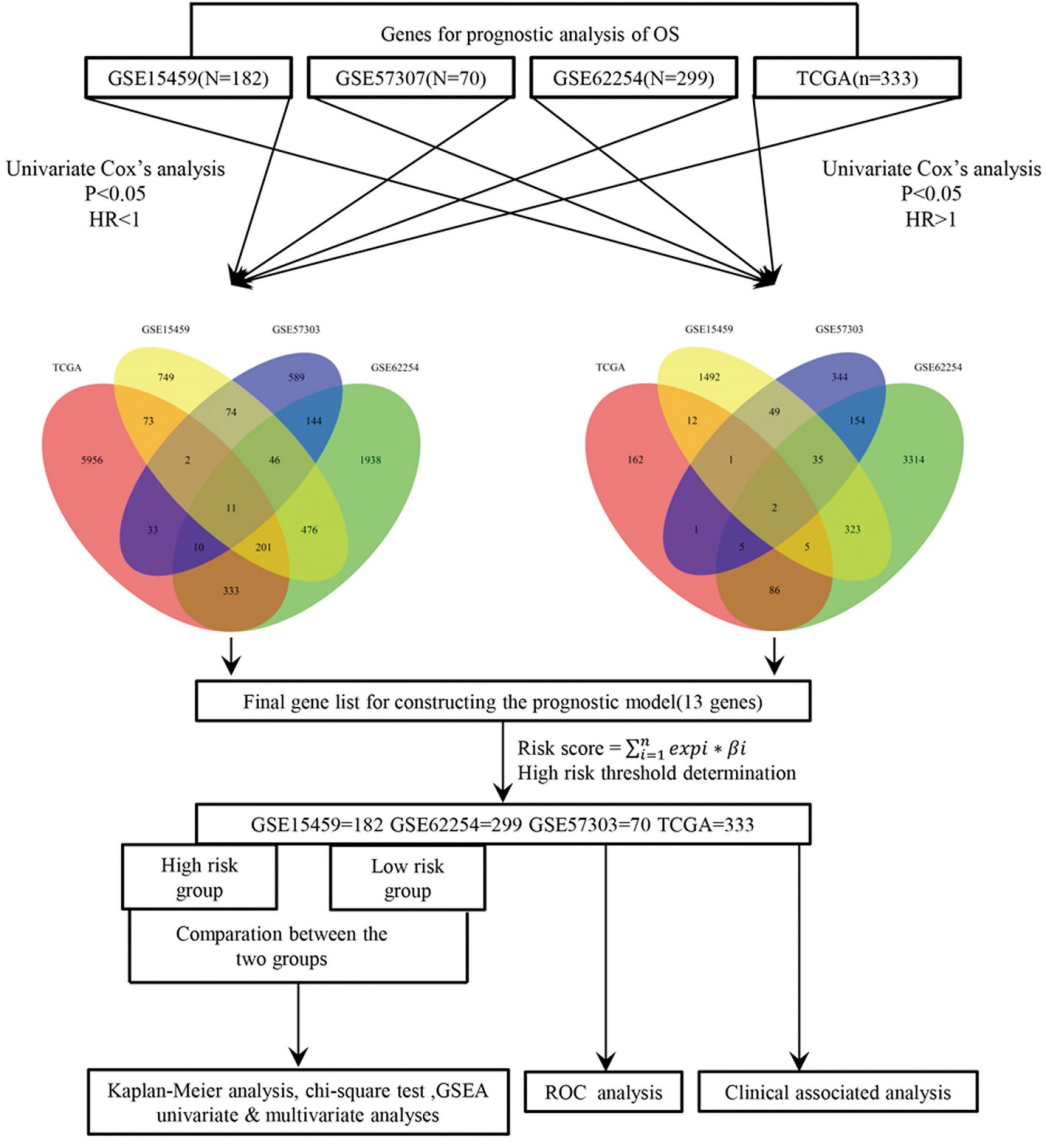
Study flow diagram.

In the present study, a univariate Cox proportional hazard regression model was used to assess the relationships between overall survival and the level of expression in each cohort. Statistical significance was assumed at p < 0.05, and the shared genes between four datasets were selected to formulate a signature model for prediction of prognosis. Risky genes (hazard ratio (HR) > 1) and protective genes (HR < 1) were defined by the HR from the univariate Cox regression analysis. Next, we used a risk score model to develop prognostic signatures, and validated it via four datasets and the entire cohort. A detailed flow‑chart of this study is depicted in Figure 1.

Based on the expression of the gene signature and corresponding weighted coefficient, we calculated a risk score for each patient as follows:


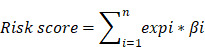


In the above equation, *n* was the number of prognostic genes, expi was the expression value of gene i, and βi was the regression coefficient of gene i. According to the median risk score obtained, patients with GC in each cohort were segmented into high-risk and low-risk group. Subsequently, we investigated the relationship between the prognosis signature and RFS based on TCGA.

### Gene set enrichment analysis (GSEA)

GSEA was implemented to explore the Kyoto Encyclopedia of Genes and Genomes (KEGG) pathways that were significantly enriched in high-risk tumor samples by GSEA v4.0.3 (http://www.broadinstitute.org/gsea/index.jsp). The gene sets of canonical pathways (c2.all.v7.0.symbols.gmt) were used 1,000 times for each analysis to obtain the normalized enrichment score (NES). P<0.05 and NES >1 were considered statistically significant.

### Statistical analysis

The data were analysed using R software (version 3.4.3). Comparisons between the two groups were inspected by the Kaplan‑Meier curve and compared by the log‑rank test. The chi-square test was implemented to evaluate the relativity between the risk score and recurrence. To investigate whether the prognostic gene signature could be independent of other clinical and pathological characteristics, we carried out univariate and multivariate Cox proportional hazard regression models. Factors with P value < 0.05 in the univariate analyses were included in the multivariate analyses 10. Unless otherwise stipulated, P < 0.05 was set as the cut‑off value. Receiver operating characteristic (ROC) curve analysis was performed to examine the predictive accuracy of the risk score for time-dependent disease outcomes within three years. Afterwards, the area under the curve (AUC) values were calculated according to the ROC curves.

## Results

### Prognostic signature generation

Survival-associated genes were identified via univariate Cox regression analysis in each dataset. Under the cut-off threshold of HR < 1 and P < 0.05, 1919 genes in GSE15459, 3924 genes in GSE62254, 591 genes in GSE57303, and 274 genes in TCGA were selected as candidate protective genes. According to the screening criteria (HR > 1 and P < 0.05), 1632 genes in GSE15459, 3159 genes in GSE62254, 909 genes in GSE57303, and 6619 genes in TCGA were selected as risky candidate genes. Finally, a total of 13 common genes (ADAT3, TMEM171, DCBLD2, MARCKS, CLIP4, CTNNAL1, PIP4K2A, ZBTB10, NRP1, CST6, PLTP, CD109, and JAZF1) were finally identified and nominated as the “13-gene signature” by overlapping these candidate genes, including 2 protective genes and 11 risky genes (Table S3). The general information of these 13 genes and the prognostic correlation of the 13 in each dataset are displayed in Tables 1 & 2.

### Thirteen-gene prognostic signature validation

**Figure 2:**
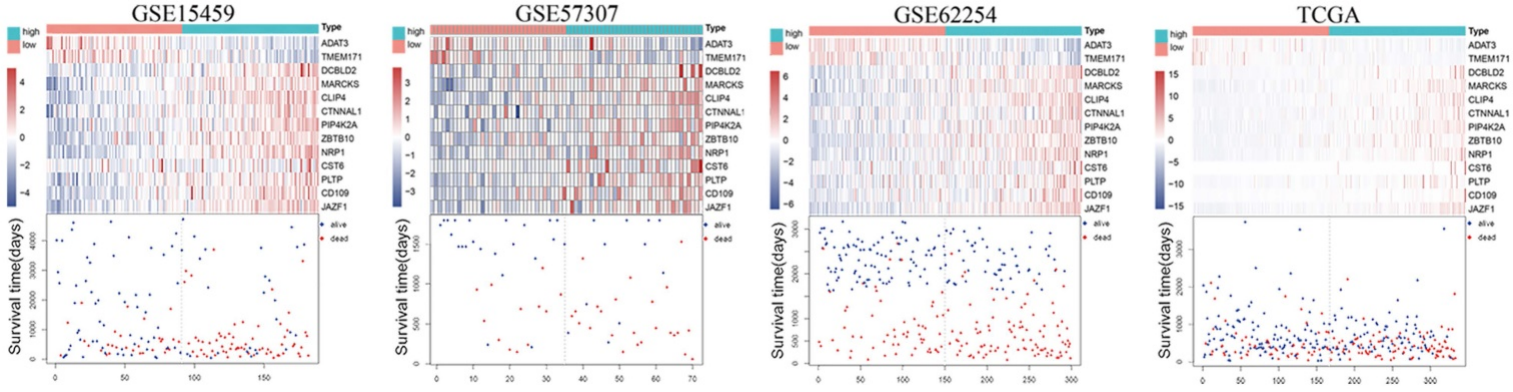
Risk-score analysis of GC patients in the four datasets. In each dataset, the gene expression profiles, and patients' survival status are displayed. The black-dotted line represents the median cut-off, dividing patients into high- and low-risk groups.

**Figure 3:**
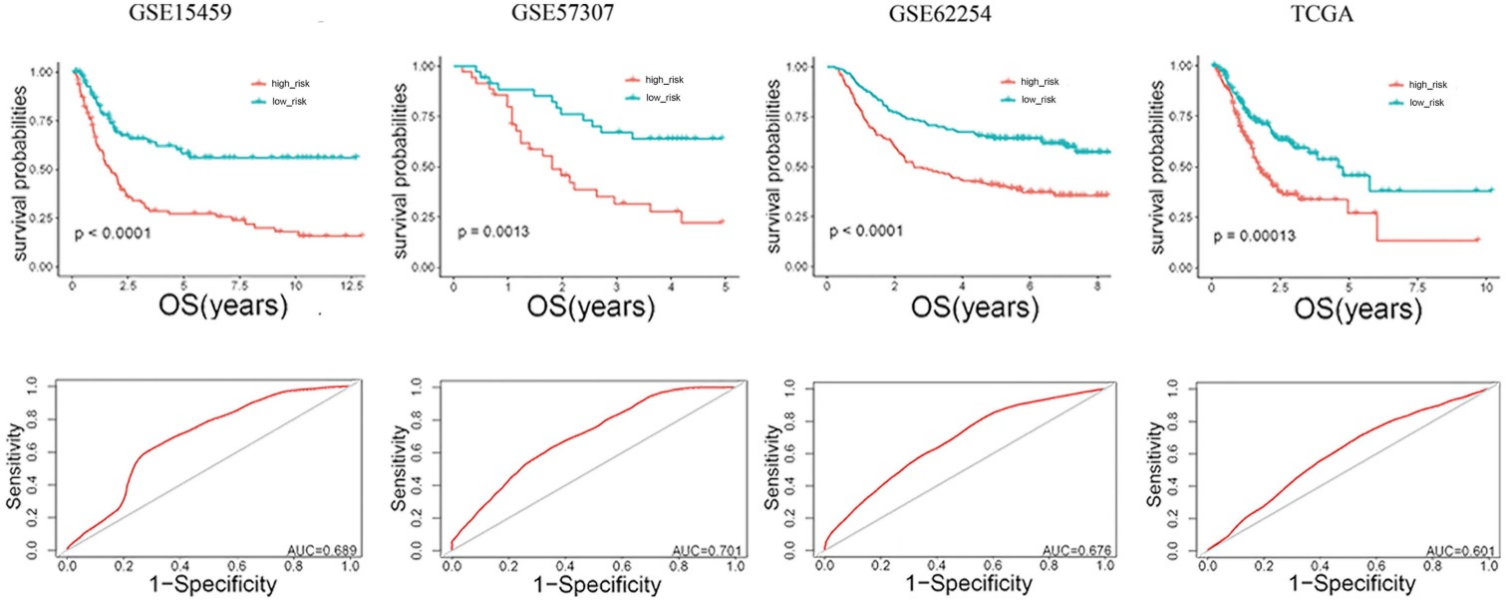
Kaplan-Meier and ROC curves for the 13-gene signature in the four datasets. Patients with high risk scores had poor outcome in terms of overall survival.

The risk score (RS) for each patient was achieved based on the expression levels of the 13-gene signature and corresponding weighted coefficients. The samples in each data set were sorted by risk score, and the median risk score was set as the cut-off point to stratify the subjects into high‑ and low‑risk groups. In Figure 2, the survival status, and gene expression of patients in each dataset were profiled, which showed that the survival events decreased as RS increased. Furthermore, the survival curves demonstrated that the prognosis of patients with a high-risk score suffered a poorer prognosis than those with a low-risk score (GSE15459: P < 0.0001, GSE57303: P = 0.0013, GSE62254: P < 0.0001, TCGA: P = 0.00012) (Figure 3). The area under the ROC curve of the 13-gene signature was 0.689, 0.701, 0.676, and 0.601 in GSE57303, GSE62254, GSE15459, and TCGA respectively, revealing that the 13-gene prognostic model had a certain accuracy in predicting outcome in GC (Figure 3). The above data confirmed that RS derived from a 13-gene signature could appropriately predict the prognosis of patients with GC.

### The 13-gene signature is an independent prognostic factor

To investigate the prognostic prediction value of the 13-gene prognostic signature and identify the independent factors in gastric cancer, univariate and multivariate Cox regression analyses were performed. Covariates are composed of RS and other clinicopathological characteristics, such as gender, age, stage, Lauren classification and recurrence. Multivariate regression analysis revealed that the 13-gene signature was significantly correlated with OS even after adjustment for other covariates in the four datasets (GSE15459: P = 9.84E-05, GSE57303: P = 2.07E-03, GSE62254: P = 4.27E-03, and TCGA: P = 4.89E-03) (Table 3) or the entire cohort (P = 2.59E-12) (Table 4).

### The prognostic signature is associated with cancer recurrence

**Figure 4:**
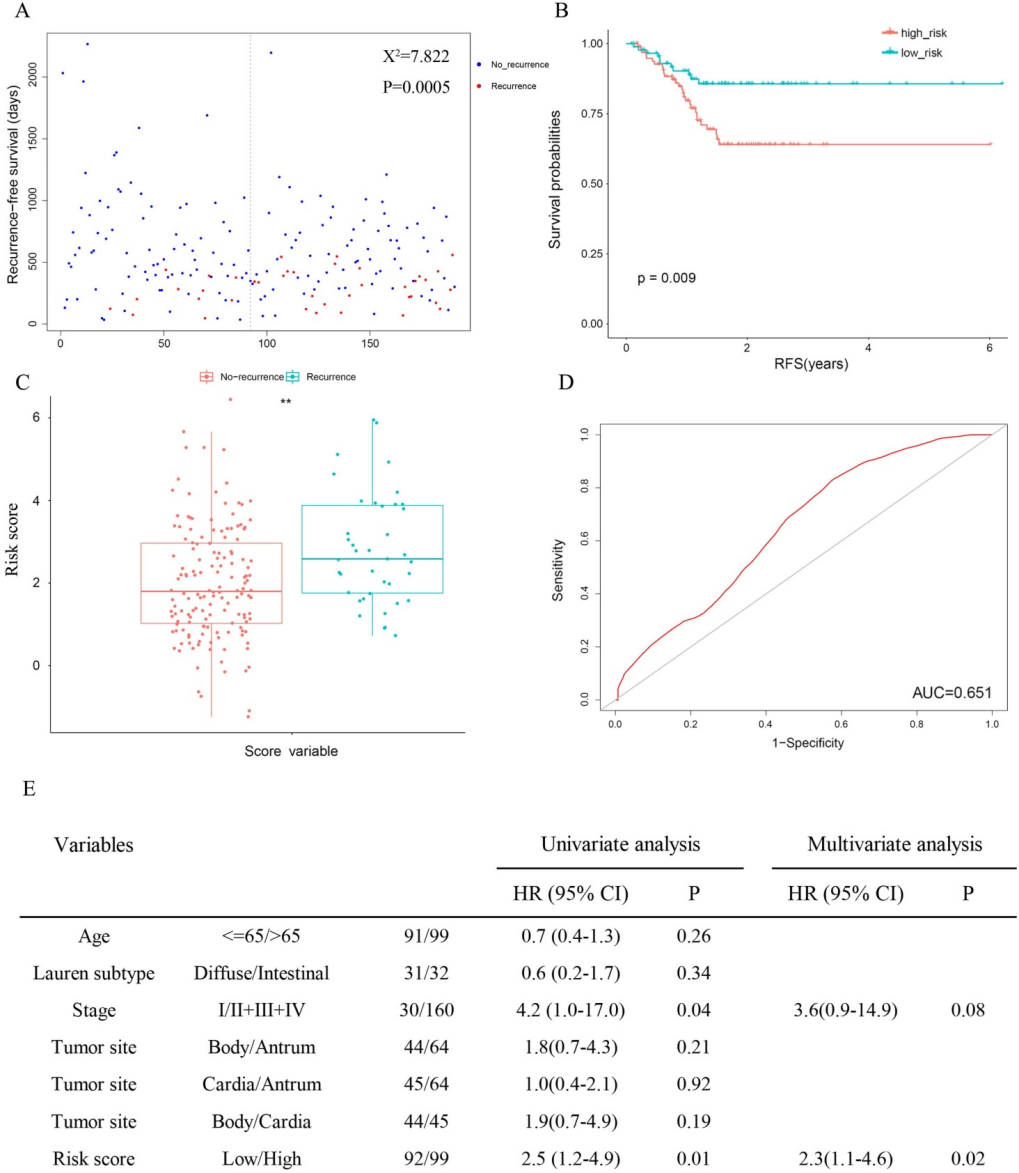
The 13-gene signature is associated with cancer recurrence.

Since cancer recurrence and RS are both independent factors of patient survival in TCGA (Table 3), the relativity between recurrence and the risk score was tested. The results revealed significantly favourable RFS in the low-risk group (Figure 4A: χ2 = 7.822, P = 0.0005; Figure 4B: P = 0.009), and patients with recurrence were more likely to have a higher risk score (Figure 4C). Meanwhile, the AUC for 3-year recurrence-free survival predictions was 0.651(Figure 4D). Then we performed univariate and multivariate Cox proportional hazards models. In univariate analyses, high stage (HR = 4.2, 95% CI = 1.0-17.0, P = 0.04) and risk score (HR = 2.5, 95% CI = 1.2-4.9, P = 0.01) were associated with shorter (RFS). Multivariate analysis indicated that risk score was the only independent prognostic factor in terms of RFS (HR = 2.3, 95% CI = 1.1-4.6, P = 0.02).

### Stratification analysis

**Figure 5:**
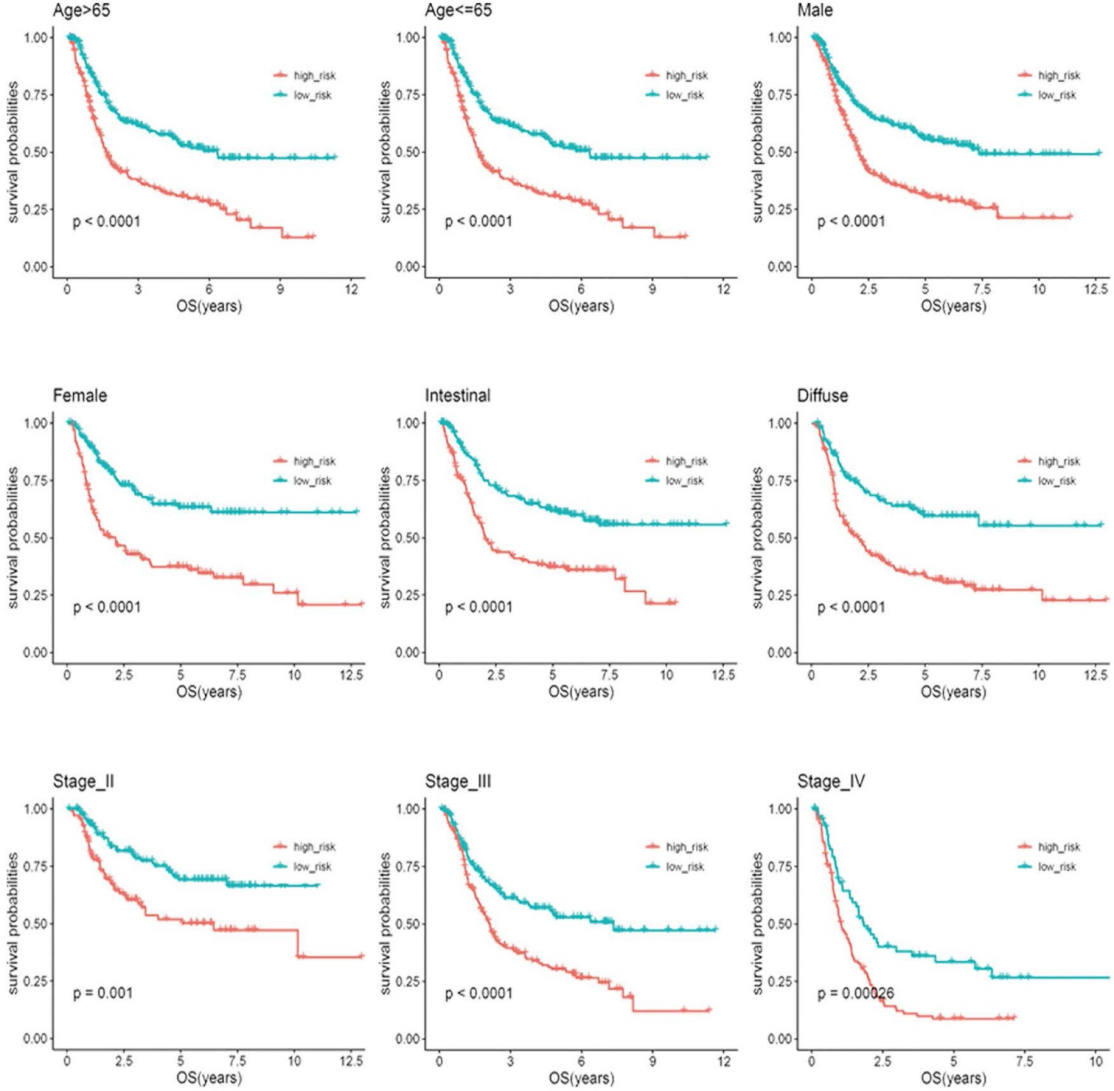
Kaplan-Meier analysis of overall survival for patients stratified by age, gender, Lauren'subtype, and stage.

Based on clinical parameters, such as age (65/>65), gender (female/male), stage (II/III/IV) and Lauren subtype (diffuse/intestinal), the patients in the entire cohort were factitiously stratified. Patients in stage I were excluded from the stratification analysis due to the small sample size. In Figure 5, the survival curves in different subgroups displayed that patients with high-risk scores in each stratum had significantly shorter OS (P < 0.05).

### Identification of KEGG pathways

**Figure 6:**
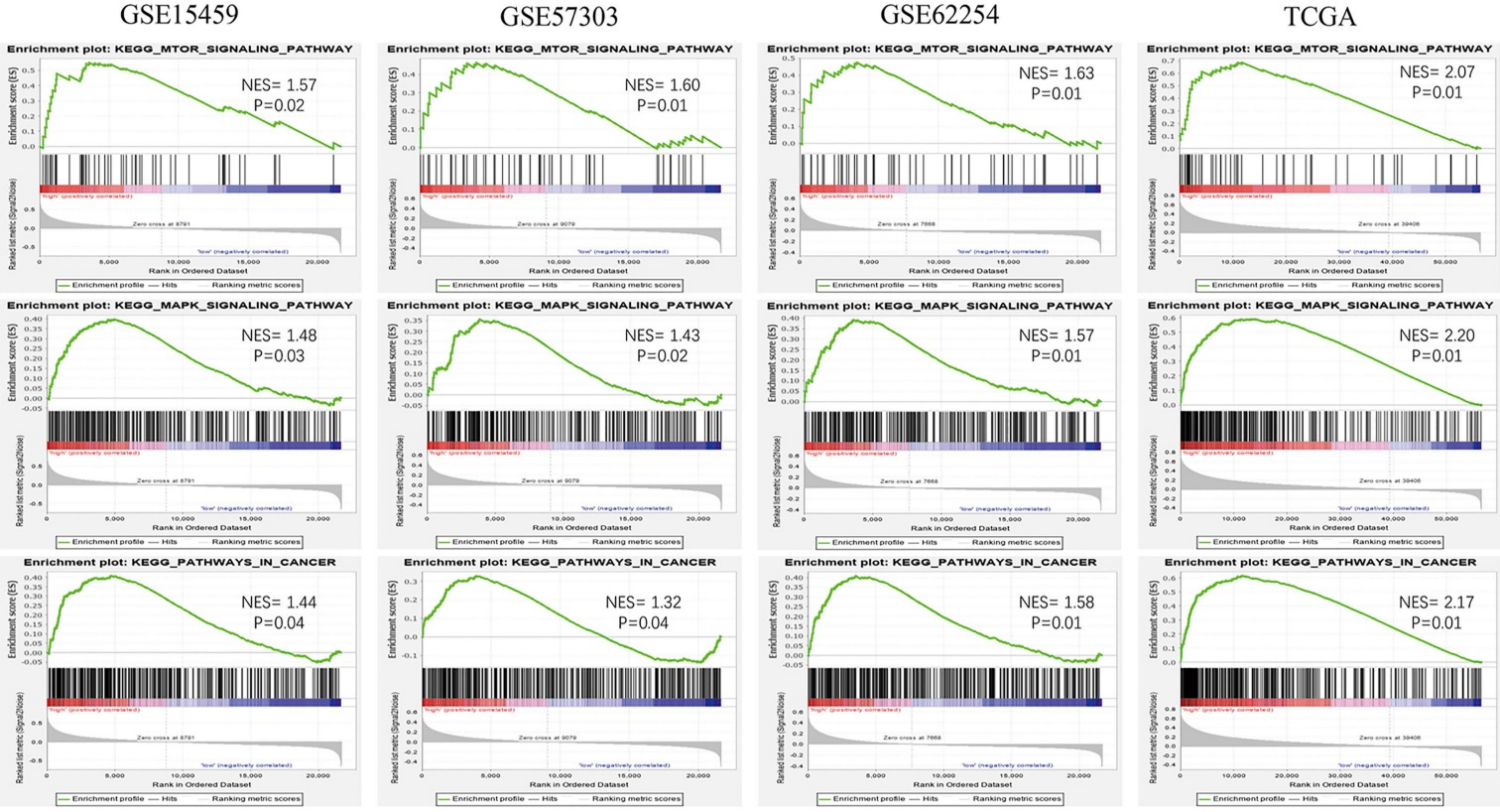
Oncological KEGG pathways enriched in the high-risk group from 4 independent datasets.

To explore the potential function of the 13-gene signature, we conducted GSEA among high-risk and low-risk patients in the four datasets. Oncological pathways, such as the mTOR signaling pathway (GSE15459: NES = 1.57, GSE57303: NES = 1.60, GSE62254: NES = 1.63, TCGA: NES = 2.07), MAPK signaling pathway (GSE15459: NES = 1.48, GSE57303: NES = 1.43, GSE62254: NES = 1.57, and TCGA: NES = 2.20, and Pathway in cancer (GSE15459: NES = 1.44, GSE57303: NES = 1.32, GSE62254: NES = 1.58, TCGA: NES = 2.17), were highly concentrated in the high-risk group (Figure 6). GSEA suggested that molecular alterations in the high-risk group were closely related to the malignant characteristics of gastric cancer.

## Discussion

Previous studies have developed a number of molecular signatures that divide patients into various prognostic groups and are involved in tumor progression 11-17. Wang et al. built a prognostic seven‐gene signature using integrated multi‐step analysis 11. Chen et al. found novel proposed clinical-immune signature based on TCGA 12. However, these assumed prognostic signatures were mostly derived from one or two training sets. In the present study, we establish 13-gene prognostic signatures by taking the intersection of the survival-related genes from four independent datasets for the first time in gastric cancer and partially handling the problems of clinical heterogeneity and insufficient sample size, which were eventually validated.

Our study suggested that the 13-gene signature was an independent prognostic factor (Tables 3 & 4). In addition, age, stage and recurrence were significantly associated with OS of patients in TCGA; stage was correlated with OS of patients in GSE15459; and stage and Lauren subtype were prominently related to OS of patients in GSE62254. Age and stage were identified as independent prognostic factors for OS in the entire patient cohort. Interestingly, Marqués-Lespier et al. indicated that diffuse-type gastric cancer is more aggressive and has a worse prognosis than intestinal-type gastric cancer 18. In our study, multivariate Cox regression analysis revealed that, except for patients in GSE62254, no significant correlation was found between the Lauren type and either OS or RFS in GC (Table 4, Figure 4E).

Among the identified 13 genes (ADAT3, TMEM171, DCBLD2, MARCKS, CLIP4, CTNNAL1, PIP4K2A, ZBTB10, NRP1, CST6, PLTP, CD109, and JAZF1) in our study, overexpression of MARCKS, CLIP4, NRP1, PLTP, CD109 has been previously associated with poor prognosis of GC 19-23. MARCKS aggravates gastric cancer tumorigenesis and progression via EMT pathway 19. It has been found that high expression of CLIP4 and CD109 was associated with poor GC overall survival according to assay and RNA-Seq data of patients with GC 20, 21. Loss of PLTP expression has been reported to inhibite the proliferation in both AGS and SGC‐7901 cell lines 23. NRP1 plays an essential role in the proliferation, migration, and invasion of gastric cancer cells 24. Our findings are consistent with these studies. In addition, ZBTB10 was regarded to regulate specificity proteins (Sp) by reactive oxygen species (ROS)- microRNA27a 25. Chen et al. proposed that JAZF1 suppresses proliferation and induces apoptosis via TAK1/NF-κB pathways 26. DCBLD2 over-expression has been implicated in causing tumorigenesis, invasion and metastasis in colorectal cancer expression 27. CTNNAL1 can contribute to drug-resistance of melanoma through the way of activating NF-κB and AP-1 28. Shin et al. believed that PIP4K2A played a negative regulatory role on PI3K in PTEN-deficient glioblastoma 29. Emerging evidence has shown that High CST6 expression predicts poor prognosis in Triple-Negative Breast Cancer 30. For ADAT3 and TMEM171, there is little published data on TMEM171 and ADAT3 function in cancer. Except for MARCKS, CLIP4, NRP1, PLTP, CD109, other mRNAs are newly reported to be associated with GC survival.

The TNM staging system, currently used as the most important and basic tool for GC patient stratification, is deficient in accurately predicting individual survival 3-5. This happens because nearly one-third of patients experienced recurrence after surgery, whereas the current staging system cannot accurately reflect it 31, which is also displayed in our study (Figure 4E). This is directly linked to chemotherapy strategy after gastrectomy. GSEA disclosed that several oncological pathways, including the mTOR signaling pathway and MAPK signaling pathway, were significantly concentrated in the high-risk group. Among them, the activated mTOR signaling pathway is critical for cell transformation, growth, metastasis and predicts poor prognosis in gastric cancer 32, 33. The consequences of increased mTOR pathway signaling can also lead to drug resistance, which continues to be the principal limiting factor to achieving cures in patients with cancer 34, 35. In trastuzumab-resistant HER2 positive gastric cancer cells, mTOR pathway is among multiple signaling pathways that mediate trastuzumab resistance 36. Notably, suppressing the mTOR pathway can significantly inhibit tumor progression and improve the efficacy of trastuzumab in GC 36, 37. The MAPK signaling pathway is reported to be frequently activated in the process of tumor development, such as proliferation, migration, and invasion 38, 39. The expression of MAPKs is elevated in almost all high-grade GC and is correlated with tumorigenesis and metastatic potential 40. Similarly, lots of studies have demonstrated that the MAPK signaling pathway has an effect on chemotherapy resistance in gastric cancer. Guo et al. found that the p38-MAPK pathway was activated in vincristine-resistant gastric cancer SGC7901/VCR cells and confirmed its regulatory effect on multidrug resistance 41. In addition, emerging evidence suggests that SB203580, a selective inhibitor of p38 MAPK, increases sensitivity to doxorubicin in two gastric cancer cell lines (SGC7901 and BGC823) by inducing apoptosis *in vitro* and *in vivo* 42. In general, these oncological pathways may provide further understanding of the risk score model in GC, and repression of these pathways might be a useful therapeutic strategy for high-risk patients.

Some flaws in our study should be taken into consideration. First, this is a retrospective study and validation of the signature for each patient in a large-scale prospective clinical trial is necessary. Second, the molecular mechanism of these genes remains unclear, and further functional experiments are needed to in the future.

## Conclusions

**Figure 7:**
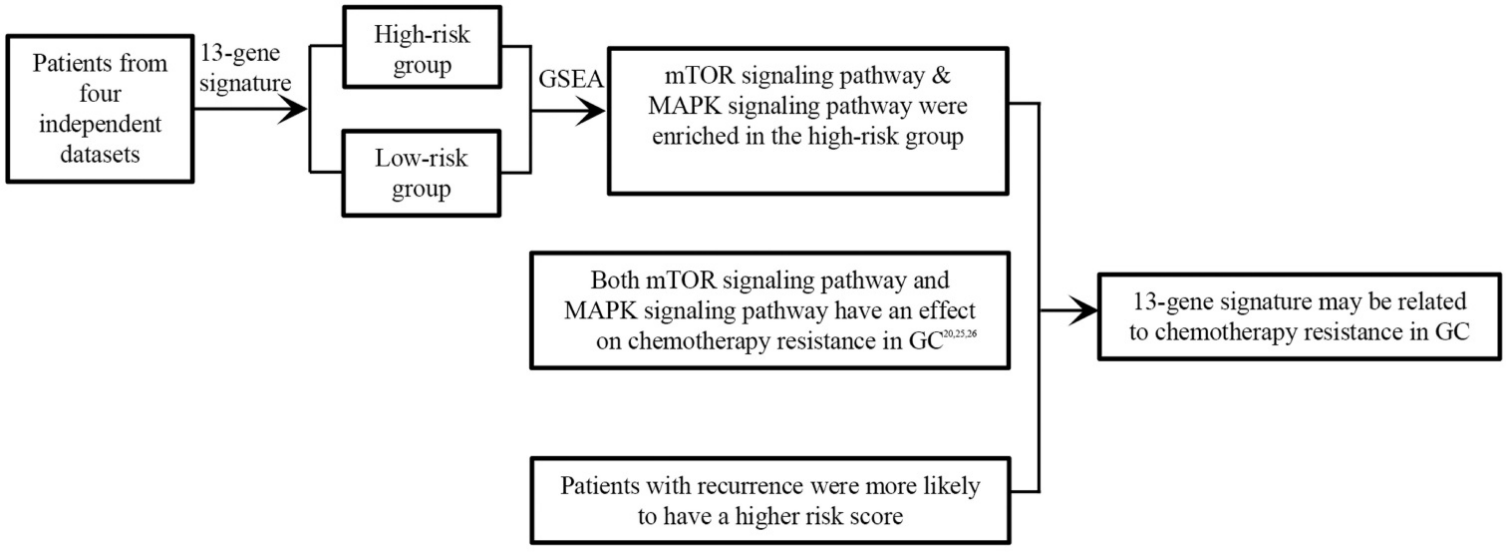
13-gene signature may be related to chemotherapy resistance in GC.

Collectively, we identified a novel 13-gene signature as a potential prognostic biomarker based on four independent datasets for the first time in GC, that was independent of other clinical factors. Besides, most of the 13 genes were the first time finding associated with the prognosis of GC. Last but not least, the signature may be associated with cancer recurrence and chemotherapy resistance in GC (Figure 7). Further experimental studies are warranted to elucidate the mechanisms of the 13 genes in gastric cancer.

## Supplementary Material

Supplementary table S1 and table S2.

Supplementary table S3.

## Figures and Tables

**Table 1:** General information of the 13 genes for constructing the prognostic signature

Gene stable ID	Gene name	Gene type	Chromosome	Gene start (bp)	Gene end (bp)
ENSG00000213638	ADAT3	Protein coding	19	1905399	1913447
ENSG00000157111	TMEM171	Protein coding	5	73120569	73131809
ENSG00000057019	DCBLD2	Protein coding	3	98795941	98901695
ENSG00000277443	MARCKS	Protein coding	6	113857345	113863475
ENSG00000115295	CLIP4	Protein coding	2	29097705	29189643
ENSG00000119326	CTNNAL1	Protein coding	9	108942569	109013522
ENSG00000150867	PIP4K2A	Protein coding	10	22534854	22714578
ENSG00000205189	ZBTB10	Protein coding	8	80485619	80526265
ENSG00000099250	NRP1	Protein coding	10	33177492	33336262
ENSG00000175315	CST6	Protein coding	11	66012008	66013505
ENSG00000100979	PLTP	Protein coding	20	45898621	45912155
ENSG00000156535	CD109	Protein coding	6	73695785	73828316
ENSG00000153814	JAZF1	Protein coding	7	27830573	28180795

**Table 2:** Univariate regression analysis of the 13 genes and overall survival of GC patients in 4 datasets

Genes	GSE15459		GSE62254		GSE57303		TCGA	
	HR (95% CI)	*P*	HR (95% CI)	*P*	HR (95% CI)	*P*	HR (95% CI)	*P*
ADAT3	0.46 (0.24-0.86)	1.52E-02	0.17 (0.07-0.40)	4.09E-05	0.04 (0.00-0.39)	5.49E-03	0.95 (0.92-0.98)	1.77E-03
CD109	1.39 (1.11-1.75)	4.73E-03	0.64 (0.52-0.80)	5.85E-05	1.59 (1.01-2.50)	4.68E-02	1.01 (1.01-1.02)	3.28E-05
CLIP4	1.34 (1.06-1.69)	1.49E-02	1.37 (1.06-1.77)	1.78E-02	2.00 (1.19-3.35)	8.93E-03	1.04 (1.01-1.07)	7.57E-03
CST6	1.19 (1.05-1.34)	4.96E-03	1.75 (1.18-2.60)	5.72E-03	1.34 (1.02-1.76)	3.76E-02	1.00 (1.00-1.00)	3.78E-04
CTNNAL1	1.35 (1.03-1.77)	2.90E-02	1.79 (1.36-2.37)	4.09E-05	2.06 (1.11-3.83)	2.22E-02	1.02 (1.01-1.03)	1.89E-03
DCBLD2	1.94 (1.31-2.87)	9.55E-04	1.67 (1.36-2.06)	1.20E-06	2.62 (1.45-4.74)	1.41E-03	1.02 (1.00-1.03)	3.85E-02
JAZF1	1.34 (1.04-1.73)	2.26E-02	1.57 (1.28-1.93)	1.46E-05	1.57 (1.00-2.46)	4.93E-02	1.03 (1.00-1.05)	1.69E-02
MARCKS	2.25 (1.44-3.53)	3.92E-04	1.22 (1.02-1.45)	2.65E-02	2.44 (1.26-4.71)	7.85E-03	1.01 (1.00-1.01)	3.20E-05
NRP1	2.38 (1.53-3.70)	1.15E-04	1.81 (1.24-2.63)	2.06E-03	2.21 (1.08-4.54)	3.00E-02	1.02 (1.01-1.03)	9.26E-05
PIP4K2A	1.54 (1.03-2.30)	3.43E-02	1.73 (1.35-2.20)	1.03E-05	3.00 (1.14-7.88)	2.55E-02	1.02 (1.00-1.03)	9.27E-03
PLTP	1.21 (1.00-1.45)	4.64E-02	1.35 (1.12-1.62)	1.32E-03	1.39 (1.01-1.91)	4.21E-02	1.00 (1.00-1.00)	3.34E-02
TMEM171	0.72 (0.53-0.98)	3.73E-02	1.25 (1.03-1.53)	2.56E-02	0.50 (0.27-0.91)	2.28E-02	0.99 (0.98-1.00)	4.90E-02
ZBTB10	1.61 (1.18-2.18)	2.58E-03	1.42 (1.00-2.00)	4.76E-02	1.81 (1.06-3.08)	2.99E-02	1.03 (1.00-1.05)	3.43E-02

HR, hazard ratio; CI, confidence interval.

**Table 3:** Univariate and multivariate Cox regression analyses of the gene signature and overall survival of GC patients in 4 independent datasets

Variables	Patients (N)	Univariate analysis		Multivariate analysis	
		HR (95% CI)	*P*	HR (95% CI)	*P*
**GSE57303**					
Age (≤65/>65)	45/23	0.6 (0.3-1.4)	2.50E-01		
Gender (Male/Female)	52/18	0.6 (0.2-1.3)	1.60E-01		
Lauren subtype (Diffuse/Intestinal)	35/20	1.1 (0.5-2.3)	8.70E-01		
Stage (I+II/III+IV)	13/57	1.5 (0.6-4.0)	3.70E-01		
Risk score (Low/High)	35/35	3.0 (1.5-6.1)	2.10E-03	3.0 (1.5-6.1)	2.07E-03
**GSE15495**					
Age (≤65/>65)	81/101	1.0 (0.6-1.4)	7.80E-01		
Gender (Male/Female)	116/66	0.7 (0.5-1.1)	1.30E-01		
Lauren subtype (Diffuse/Intestinal)	73/91	0.9 (0.6-1.3)	5.40E-01		
Stage (I+II/III+IV)	59/123	6.5 (3.6-12)	6.40E-10	6.2 (3.4-11.2)	2.69E-09
Risk score (Low/High)	91/91	2.7 (1.7-4.1)	9.20E-06	2.4 (1.5-3.7)	9.84E-05
**GSE62254**					
Age (≤65/>65)	171/128	1.3 (1.0-1.8)	7.90E-02		
Gender (Male/Female)	198/101	1.1 (0.8-1.6)	5.20E-01		
Lauren subtype (Diffuse/Intestinal)	124/140	0.6 (0.4-0.8)	3.40E-03	0.9 (0.6-1.2)	4.12E-01
Stage (I+II/III+IV)	125/172	3.5 (2.4-5.1)	8.20E-11	3.5 (2.3-5.4)	1.36E-08
Risk score (Low/High)	150/149	2.1 (1.5-2.9)	1.40E-05	1.7 (1.2-2.5)	4.27E-03
**TCGA**					
Age (≤65/>65)	152/178	1.6 (1.1-2.2)	1.10E-02	1.7 (1.1-2.7)	2.55E-02
Gender (Male/Female)	216/117	0.8 (0.5-1.1)	1.20E-01		
Stage (I+II/III+IV)	151/168	1.8 (1.2-2.5)	1.80E-03	1.3 (0.8-2.0)	2.85E-01
Lauren subtype (Diffuse/Intestinal)	58/70	1.3 (0.8-2.2)	3.30E-01		
Recurrence (No/Yes)	209/57	3.7 (2.4-5.7)	2.40E-09	3.6 (2.3-5.7)	3.04E-08
Risk score (Low/High)	167/166	1.9 (1.4-2.7)	1.70E-04	2.0 (1.2-3.3)	4.89E-03

HR, hazard ratio.

**Table 4:** Univariate and multivariate Cox regression analyses of the gene signature and overall survival of GC patients in entire cohort

Variables	Patients (N)	Univariate analysis		Multivariate analysis	
		HR (95% CI)	*P*	HR (95% CI)	*P*
Age (≤65/>65)	449/430	1.3 (1.0-1.5)	1.40E-02	1.6 (1.3-1.9)	9.10E-06
Gender (Male/Female)	582/302	0.9 (0.7-1.0)	1.30E-01		
Lauren subtype (Diffuse/Intestinal)	300/326	0.8 (0.6-1.0)	5.50E-02		
Stage (I+II/III+IV)	348/520	2.9 (2.3-3.7)	2.60E-20	2.9 (2.3-3.6)	2.00E-16
Risk score (Low/High)	443/441	2.2 (1.8-2.7)	2.30E-14	2.1 (1.7-2.6)	2.59E-12

HR, hazard ratio; CI, confidence interval.
