# Supplementary material for: The prediction of survival in Gastric Cancer based on a Robust 13-Gene Signature
Source: J Cancer. 2021 Apr 12;12(11):3344–53. doi: 10.7150/jca.49658 (PMC8100809; doi:10.7150/jca.49658)
Supplement: Supplementary file 1 — Supplementary table S1 and table S2. [file jcav12p3344s1.pdf]

Table S1. Detailed clinic parameters for 884 patients

Variables	GSE57303	GSE15459	GSE62254	TCGA
Age				
Mean(media)	60.7(63)	64.4(66)	62.0(64)	65.2(67)
IQR	55-68	57-73	55-70	58-72
Gender				
Male	52	116	198	216
Female	18	66	101	117
Lauren subtype				
Diffuse	35	73	124	58
Intestinal	20	91	140	70
Mixed	15	18	16	0
NA	0	0	19	205
Stage				
I	1	31	30	44
II	12	28	95	107
III	45	66	95	136
IV	12	57	77	32
NA	0	0	2	14
Overall survival				
Mean(media)	975.9(900)	1215.4(609)	1522.9(1742)	640.4(486)
IQR	397.5-1500	296.8-1909.8	542-2377	294-819
Tumor site				
Body	NA	NA	107	80
Antrum	NA	NA	154	81
Cardia	NA	NA	32	120
Fundus	NA	NA	0	39
Lesser curvature of stomach	NA	NA	0	1
Whole	NA	NA	6	12

IQR, interquartile range

Table S2. Detailed clinic parameters for 191 patients

Variables	TCGA
Age	
Mean(media)	64.9(66)
IQR	58-72
Gender	
Male	116
Female	75
Lauren subtype	
Diffuse	31
Intestinal	32
NA	128
Stage	
I	30
II	75
III	73
IV	12
NA	1
Tumor site	
Body	44
Antrum	64
Cardia	45
Fundus	31
NA	7
Recurrence-free survival	
Mean(media)	553.0(451)
IQR	281-737.5

IQR, interquartile range
